# Demographic and clinical characteristics of patients with eyelid eczema attended at a referral service from 2004 to 2018^☆^

**DOI:** 10.1016/j.abd.2021.10.014

**Published:** 2022-11-02

**Authors:** Mariana de Figueiredo Silva Hafner, Victoria Cerqueira Elia, Rosana Lazzarini, Ida Duarte

**Affiliations:** a Dermatology Clinic, Hospital da Santa Casa de Misericórdia de São Paulo, São Paulo, SP, Brazil; b Faculty of Medical Sciences, Santa Casa de São Paulo, São Paulo, SP, Brazil

Dear Editor,

Among the etiologies of eyelid eczema, allergic contact dermatitis (ACD) is the most frequent one. The allergens, in these cases, can be present in products applied directly to the eyelids (e.g.: eye drops), to other areas, such as the scalp (e.g.: shampoos) and nails (e.g.: nail polish), or even dispersed in the environment (e.g.: perfumes acting as aeroallergens).^1^, ^2^, ^3^, ^4^, ^5^

There are few Brazilian studies on the subject and thus, the importance of the present study, which aimed to determine demographic and clinical characteristics of patients with eyelid eczema undergoing patch testing at a reference service between 2004 and 2018, their etiologies and allergens.

Data from medical records of patients with eyelid eczema who were submitted to patch testing during the study period were retrospectively assessed. These tests followed the application and reading methodology recommended by the International Contact Dermatitis Research Group. According to the anamnesis, the allergens used in the tests were: Brazilian standard battery (30 substances/FDA-Allergenic, RJ, Brazil), cosmetics (10 substances/FDA-Allergenic, RJ, Brazil), Latin American battery (24 substances/Chemotechnique Diagnostics, Malmo, Sweden), also called regional or extended standard, hair battery (15 substances/IPI ASAC, SP, Brazil) and, when possible, individual allergens and the patient's own products. The Finn Chamber (Smart Practice, USA) or Allergo Chamber (Neoflex, São Paulo, Brazil) were used in the tests.

The study included 228 patients, 204 (89.5%) of which were females and 24 (10.5%) males. The higher proportion of women is consistent with the literature and justified by the higher use of cosmetic products that cause palpebral ACD in the female population.^6^, ^7^

The mean age of patients was 45 years. Regarding ethnicity, 114 (50%) were white, 42 black (18%), 68 were brown (30%), and 4 were yellow (1.75%). Regarding atopy, 91 (39.9%) had a personal history and 66 (28.9%) had a family history of atopy. The time lenght of the dermatosis evolution had a median value of 12 months.

The final diagnosis of the analyzed patients was ACD in 139 cases (61%), atopic dermatitis (AD) in 29 (12.7%), unclear in 28 (12.3%), irritant contact dermatitis in 18 (7, 9%), overlapping of ACD and AD in seven (3.1%) and other diagnoses in seven (3.1%).

Regarding the clinical aspects, 148 (64.5%) had eczema lesions in other regions of the body in addition to the eyelids, such as other areas of the face in 118 (51.8%), arms in 82 (36%), hands in 52 (22.8%), legs in 47 (20.6%), trunk in 43 (18.9%) and scalp in 24 (10.5%). The higher proportion of involvement in other areas of the face and upper limbs can be explained by the greater use of cosmetic products in these regions (Figure 1, Figure 2).

**Figure 1:**
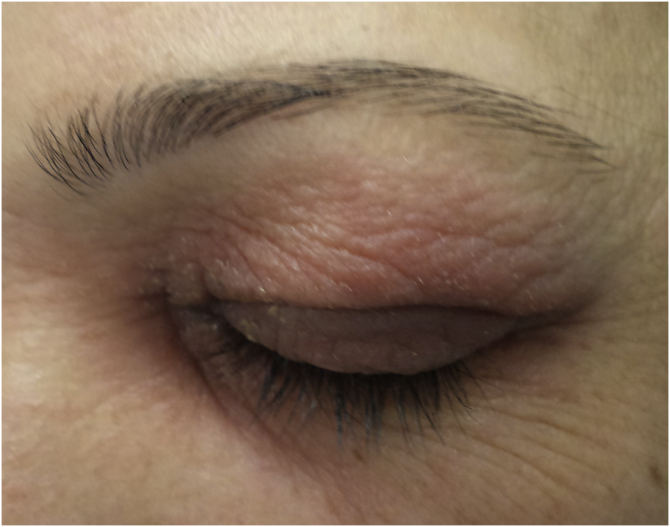
Allergic contact dermatitis caused by Kathon CG found in cosmetics.

**Figure 2:**
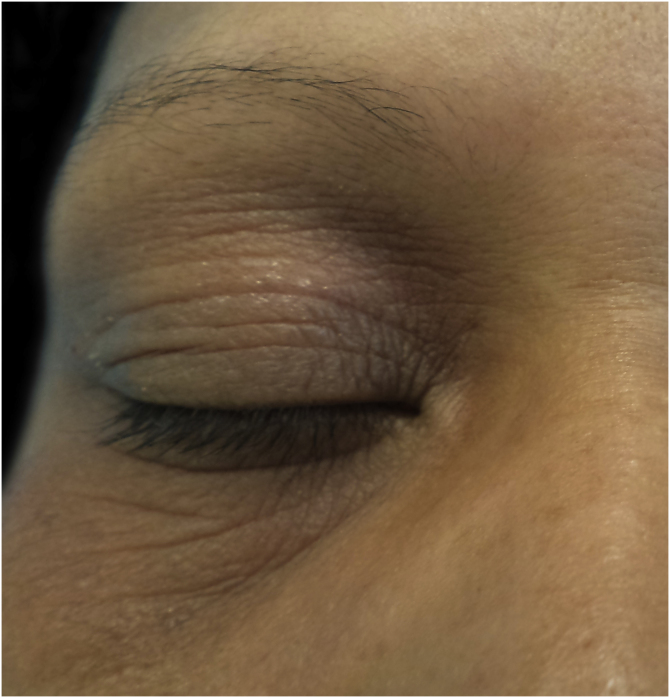
Allergic contact dermatitis caused by toluene sulfonamide resin present in nail polish.

Of the 228 cases, 183 (80.3%) had at least one positive patch test. However, after establishing the relevance, this number decreased to 147 patients (64.4%), of which 94 (41.2%) had just one relevant positive result, 31 (13.6%) had two, and 22 (9 .6%) had three or more. In a similar French study; 56.4% had at least one relevant positive test result.^7^

The use of the Brazilian standard and cosmetics batteries in patch tests made it possible to diagnose the vast majority of ACD cases (84.3%). However, it is noteworthy that the use of other batteries was adopted in the service only from 2014 onwards.

Among the patients with a final diagnosis of ACD, the main etiologies were nail polish in 53 (36%), topical medications in 40 (27.2%), unspecified cosmetics in 36 (24.5%), hair dyes in 20 (13.6%), metals in 23 (15.6%), rubber in ten (6.8%) and shampoos in six (4%). The relevant allergens are shown in Table 1.

**Table 1:** Relevant allergens found in patch tests performed on patients with eyelid eczema.

Allergen	Quantity	Frequency of positivity
Toluene sulfonamide formaldehyde resin	52	33.76%
Paraphenylenediamine	21	9.21%
Nickel sulfate	19	8.33%
Perfume mix 1	16	7.00%
Neomycin	15	6.57%
Kathon/Methylisothiazolinone	15	6.57%
Formaldehyde	12	5.26%
Promethazine	9	3.94%
Tiuram mix	8	3.50%
Ethylenediamine	6	2.63%
Carba mix	6	2.63%
Lanolin	5	2.19%
Potassium bichromate	5	2.10%
Ketoconazole	4	NA
Balm from Peru	4	1.70%
Epoxy resin	3	1.31%
Quinoline-mix	3	1.31%
Propylene glycol	3	1.31%
Colophony	3	1.31%
Cocoamidopropyl betaine	3	11.11%
P-aminophenol	2	NA
Lauryl glucoside	2	NA
Patient's nail polish	2	NA
Budesonide	2	7.40%
Benzocaine	2	0.87%
Triethanolamine	1	0.64%
Tixocortol pivalate	1	3.70%
Perfume mix 2	1	3.70%
Palladium	1	3.70%
M-aminophenol	1	NA
Thiomorpholine	1	NA
Irgasan	1	0.43%
Decyl glucoside	1	NA
Patient’s eye drops	1	NA

NA, Not Applicable (tests in which allergens/single substances were eventually used, which are not routinely tested in other patients, thus not being possible to determine the frequency of positivity of these allergens/substances).

Literature studies show that cosmetics are the main cause of eyelid ACD, most often due to fragrances and preservatives, with the main allergens being: sodium gold thiosulfate, perfume mix, balsam of Peru, and nickel sulfate.^6^, ^8^ Thus, the present sample is compatible with the literature, with cosmetics also being the main etiology.

The toluene-sulfonamide-formaldehyde resin present in nail polish was the most common relevant allergen among those analyzed. It is a substance responsible for giving resistance and shine to nail polish. As the habit of using nail polish is common in our country, this allergen causes sensitization in a significant portion of the population, with values ​​higher than those found in other countries.^6^, ^9^

Another common allergen among ACD cases was paraphenylenediamine, found in most permanent hair dyes. Interestingly, only seven of the 21 patients with positive tests relevant to this allergen had concomitant lesions on the scalp, showing that patients with ACD caused by hair dye may present with eczema on the eyelids only, other areas of the face, and the cervical region.^10^

The preservatives Kathon CG and formaldehyde as well as fragrances are also observed on sensitizers present in cosmetic products in cases of eyelid ACD.^6^, ^8^, ^9^

The allergen with the highest exclude positivity in the analyzed tests was nickel sulfate, although its frequency was lower among the relevant positive tests (15.6% of eyelid ACD cases). They can be a cause of eyelid ACD due to their presence in metal products (e.g., eyelash curlers) and as makeup contaminants. The accidental transfer of nickel on contact from the hands to the eyelids is also common.^8^, ^9^

Regarding topical medications, the main allergens were neomycin and promethazine. These can cause ACD by direct contact (use of eye drops or accidental contact after application to another area) or by distant sensitization.^6^

Seven of the cases were of occupational origin: three manicurists (caused by toluene-sulfonamide-formaldehyde resin from nail polishes and nickel sulfate from metallic instruments); one masseuse (caused by Kathon present in the massage lotion); one mason (caused by cement potassium bichromate and carba/thiuram-mix of rubber gloves); one installer of aluminum frames and one-floor installer (in both cases caused by the epoxy resin of the glues and grout).

The main limitations of the study were the use of different complementary batteries and two types of chambers in the tests, which configure the non-homogeneity of technical instruments in the evaluation.

Cases related to the use of cosmetics prevailed in the study, with emphasis on nail polish, although other causes were observed. Thus, when treating patients with eyelid eczema, the investigation with patch tests is essential.

## Financial support

None declared.

## Authors' contributions

Mariana de Figueiredo Silva Hafner: Design and planning of the study; drafting and editing of the manuscript; collection, analysis, and interpretation of data; effective participation in research orientation; intellectual participation in the propaedeutic and/or therapeutic conduct of the studied cases; critical review of the literature; critical review of the manuscript; approval of the final version of the manuscript.

Victoria Cerqueira Elia: Drafting and editing of the manuscript; collection, analysis, and interpretation of data; approval of the final version of the manuscript.

Rosana Lazzarini: Drafting and editing of the manuscript; collection, analysis, and interpretation of data; effective participation in research orientation; intellectual participation in the propaedeutic and/or therapeutic conduct of the studied cases; critical review of the literature; critical review of the manuscript; approval of the final version of the manuscript.

Ida Alzira Gomes Duarte: Design and planning of the study; drafting and editing of the manuscript; collection, analysis, and interpretation of data; effective participation in research orientation; intellectual participation in the propaedeutic and/or therapeutic conduct of the studied cases; critical review of the literature; critical review of the manuscript; approval of the final version of the manuscript.

## Conflicts of interest

None declared.
